# P-Rex1 Cooperates with PDGFRβ to Drive Cellular Migration in 3D Microenvironments

**DOI:** 10.1371/journal.pone.0053982

**Published:** 2013-01-30

**Authors:** Andrew D. Campbell, Samuel Lawn, Lynn C. McGarry, Heidi C. Welch, Bradford W. Ozanne, Jim C. Norman

**Affiliations:** 1 Beatson Institute for Cancer Research, Bearsden, Glasgow, United Kingdom; 2 The Babraham Institute, Babraham Research Campus, Cambridge, United Kingdom; IDI, Istituto Dermopatico dell'Immacolata, Italy

## Abstract

Expression of the Rac-guanine nucleotide exchange factor (RacGEF), P-Rex1 is a key determinant of progression to metastasis in a number of human cancers. In accordance with this proposed role in cancer cell invasion and metastasis, we find that ectopic expression of P-Rex1 in an immortalised human fibroblast cell line is sufficient to drive multiple migratory and invasive phenotypes. The invasive phenotype is greatly enhanced by the presence of a gradient of serum or platelet-derived growth factor, and is dependent upon the expression of functional PDGF receptor β. Consistently, the invasiveness of WM852 melanoma cells, which endogenously express P-Rex1 and PDGFRβ, is opposed by siRNA of either of these proteins. Furthermore, the current model of P-Rex1 activation is advanced through demonstration of P-Rex1 and PDGFRβ as components of the same macromolecular complex. These data suggest that P-Rex1 has an influence on physiological migratory processes, such as invasion of cancer cells, both through effects upon classical Rac1-driven motility and a novel association with RTK signalling complexes.

## Introduction

The ability of tumour cells to escape their local environment and form distant metastases is a hallmark of cancer [1], [2]. Key players in this process are the RhoGTPase family of molecules, which have long been associated with the regulation of cancer cell morphology, motility and invasion [3]. Unlike related members of the RasGTPase family, reports of activating mutations of RhoGTPases, such as Rac1, RhoA or Cdc42 are relatively rare in cancer. Nonetheless, there is ample evidence for their altered function, often through aberrant activity of regulatory molecules such as RhoGEFs (guanine nucleotide exchange factors), some of which were originally isolated as transforming factors in cancer cells [4], [5].

P-Rex1 (PI(3,4,5)P_3_-dependent Rac exchanger 1) is a Rac-specific member of the *Dbl* family of RhoGEFs – multidomain proteins which catalyse dissociation of GDP from inactive RhoGTPase molecules, and facilitate activation through loading with free intracellular GTP [6], [7]. The presence of approximately 60 independent *Dbl* family GEFs permits intricate spatial and temporal regulation of the RhoGTPases [7]. Further complexity is added by upstream regulators of GEF activity, such as P-Rex1, whose activity is in turn synergistically stimulated by interaction with the phospholipid PI(3,4,5)P_3_ and βγ subunits of the heterotrimeric G-proteins [6], [8], [9]. Since the oncogenic capacity of the *Dbl* gene product was isolated and shown to mediate GEF enzymatic activity for the RhoGTPase Cdc42 [10], numerous GEFs have been implicated in the progression of cancer. Specifically, expression of P-Rex1 itself has been identified as important factor in tumour cell invasion and metastasis in a number of cancer models, both *in vivo* and *in vitro* [11]–[14].

Regulation of P-Rex1 through association with PI(3,4,5)P_3_implicates upstream signalling pathways involving receptor tyrosine kinases (RTKs), phosphatidyl inositol 3-kinase (PI3K) and PTEN in P-Rex1 driven Rac1 activity [15]. Indeed, it has previously been demonstrated that generation of PI(3,4,5)P_3_ by PI3K downstream of RTK activation can result in increased P-Rex1 activity [8], [13]. In this report, we elucidate an additional regulatory mechanism, where cooperation between P-Rex1 and the PDGFRβ receptor tyrosine kinase is both necessary and sufficient to drive migration of fibroblasts in 3D models of cellular invasion. Given that expression of P-Rex1 appears to be a key determinant in formation of distant metastases in a number of models, including melanoma, breast and prostate cancer, we propose that in certain contexts PDGFRβ and P-Rex1 may act as a novel, pro-invasive signalling module.

## Results

### P-Rex1 Overexpression Drives Morphological Changes in a Human Fibroblast Cell Line

**Figure 1:**
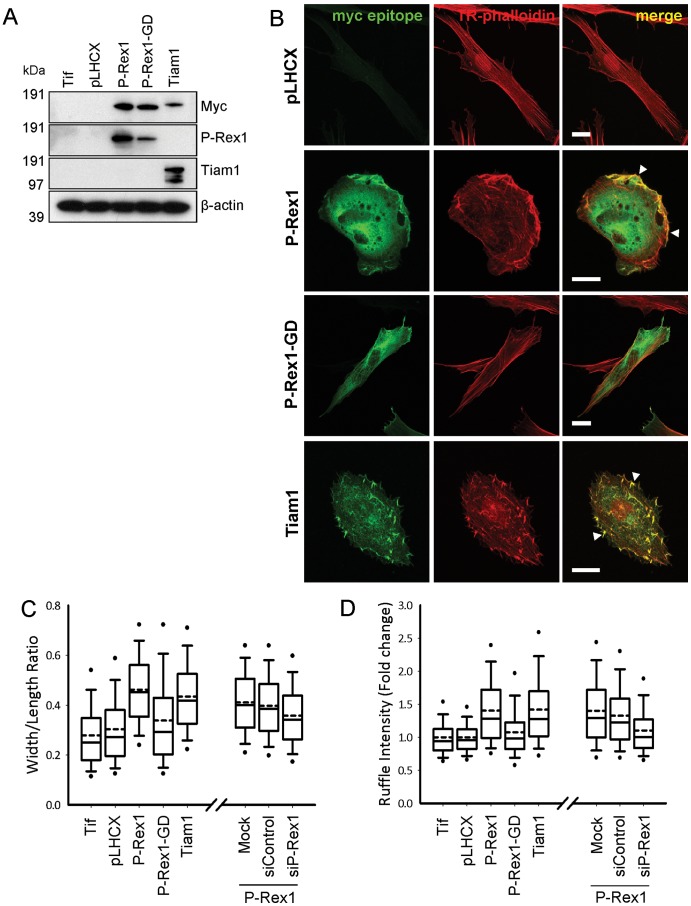
RacGEF expression alters the morphology of immortalised human fibroblasts. (A,B) Parental (Tif), and control vector (pLHCX) or RacGEF expressing telomerase-immortalised human fibroblasts were subject to immunofluorescent labelling with myc-epitope specific primary monoclonal and alexafluor-488 conjugated secondary antibodies, and counterstained with TRITC conjugated phalloidin (B). Serial optical sections were captured through confocal microscopy, and these images combined to form a 2D projection of fluorescence intensity throughout the cell. Expression of the myc-tagged RacGEF constructs was confirmed by immunoblotting with both myc-epitope and target-specific primary antibodies (A). (C,D) Control vector (pLHCX) or RacGEF expressing fibroblasts were stained as for (B), and the mean cellular width/length ratio (C) and intensity of cellular dorsal ruffling (D) was determined by means of high-throughput imaging of a sample set of more than 7,500 cells per condition. The same criteria were used to quantify the effect of P-Rex1 knockdown in P-Rex1 stable expressors (Bars 20 µm, ***P<0.0005, Mann-Whitney rank sum test).

Stable, retroviral expression of myc-epitope tagged guanine nucleotide exchange factor (GEF) constructs in an immortalised human fibroblast cell line was readily achieved and could be detected by immunoblotting for either P-Rex1 or the appropriate epitope tag (Figure 1A). This exogenous expression of P-Rex1 generated a flattened and rounded morphology, characterised by dramatic formation of lamellipodia and dorsal membrane ruffles (Figure 1B). Stable exogenous expression of Tiam1, a related GEF of the Dbl family, to similar levels as P-Rex1 (Figure 1A) resulted in similar morphological changes (Figure 1B). In both P-Rex1 and Tiam1 expressing cells, the observed ruffling morphology was very similar to that previously reported upon expression of Rac1^G12V^, a constitutively active mutant of the small GTPase molecule, Rac1 [16]. There was a concurrent reduction in the intensity of actin based stress fibres in favour of peripheral actin-rich structures such as lamellipodia and dorsal ruffles, in P-Rex1 and Tiam1 expressing fibroblasts, as indicated by TRITC-phalloidin staining of filamentous actin (Figure 1B, arrowheads). In contrast, fibroblasts expressing a control vector or a line expressing a GEF-dead mutant of P-Rex1incapable of catalysing GTP exchange (E56A, N238A – known henceforth as P-Rex1-GD) [9], exhibited no appreciable morphological changes when compared to the parental line (Figure 1B).

The morphological changes associated with GEF expression were quantified using automated, high-throughput imaging. Stable expression of P-Rex1 or Tiam1 resulted in a statistically significant 1.5 fold increase in mean width to length ratio of the cells compared to vector control over a sample set of more than 7,500 cells per condition (Figure 1C). These results were mirrored by quantification of a second independent criterion, dorsal ruffling, with its mean intensity increasing by 1.4 fold in both P-Rex1 and Tiam1 expressing cells, when compared to vector control (Figure 1D). Importantly, expression of P-Rex1-GD did not alter either of these quantitative indices of cell morphology, by comparison to vector control (Figure 1C, D). Furthermore, it was noted that the morphological changes observed upon stable, ectopic expression of P-Rex1 in this cell culture model were dependent upon P-Rex1 expression, and could be reversed through transfection with a P-Rex1-specific siRNA oligonucleotide, though not through mock treatment or transfection with a non-targetting siRNA oligonucleotide (Figure 1C, D).

### Expression of P-Rex1 Influences the Migration of Human Fibroblasts on 2D Substrates and in 3D Microenvironments

**Figure 2:**
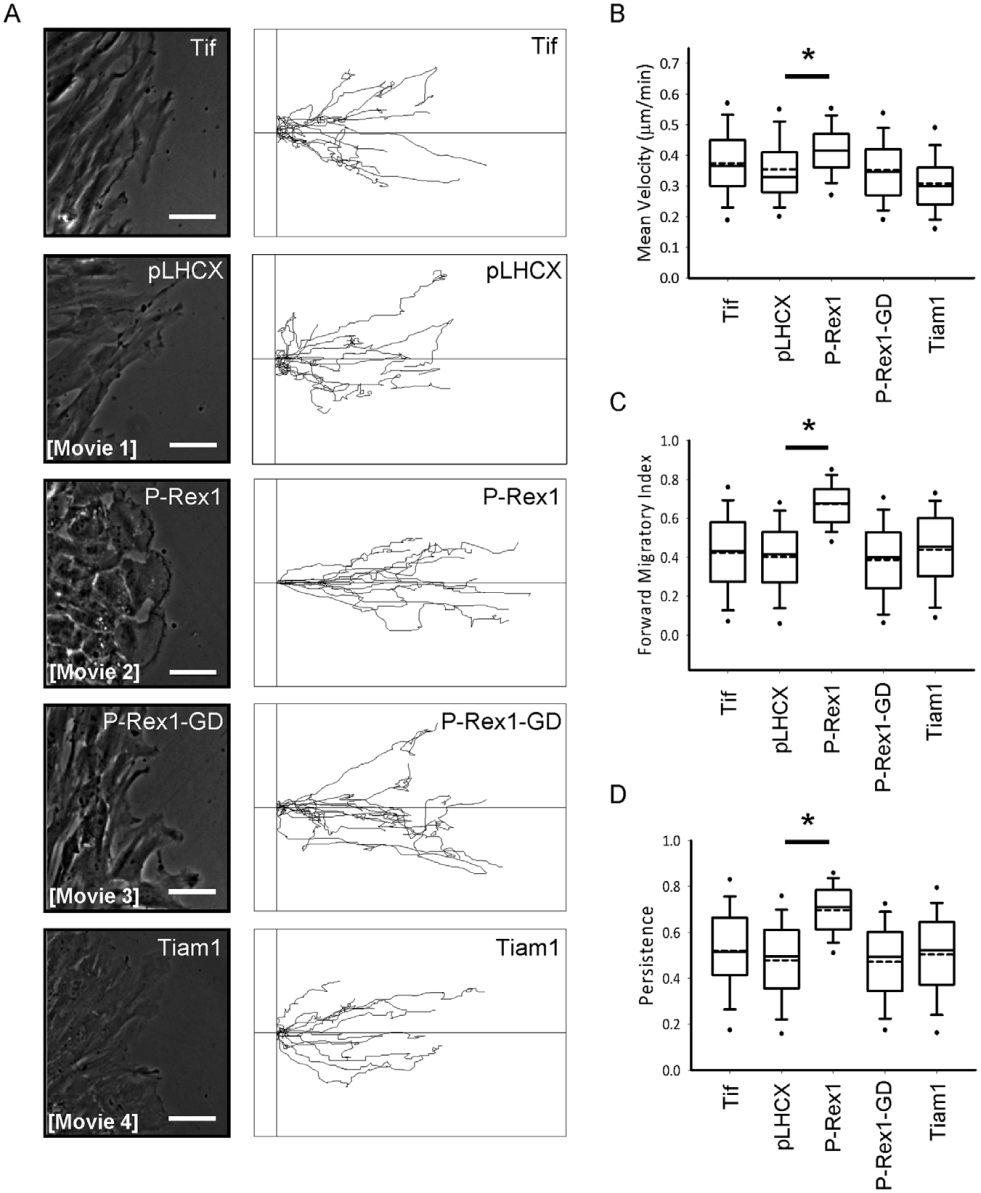
P-Rex1 expression promotes persistent migration on a 2D substrate. (A) Parental (Tif), and control vector (pLHCX) or RacGEF expressing fibroblasts emerging from the migratory front of scratch-wound assays were analysed using time-lapse microscopy, with images captured every 10 minutes over a period of 18 hours. Individual cells were manually tracked and the migratory path of representative cells depicted as track plots. The phase contrast images correspond to the initial frame from the supplementary movies S1–S4 as indicated. (B-D) Influence of RacGEF expression on migratory behaviour was quantified by determination of mean cellular velocity (B), forward migratory index (C) and migratory persistence (D). (Bars 50 µm, **P<0.005, Mann-Whitney rank sum test).

As modulation of RhoGTPase activity is commonly associated with altered cellular motility, we measured the migration of GEF-expressing or control fibroblasts initially on a 2D substrate. Cells were grown to confluence, the monolayer wounded using a 200 µl pipette tip and migration into the wound monitored by digital time-lapse microscopy in combination with live cell tracking (Figure 2A). Expression of P-Rex1 was associated with extension of large polarised lamellipodia and extensive dorsal ruffling at the leading edge, which were not observed in Tiam1 expressing fibroblasts (Figure 2A, Supplemental movies S1–S4). Quantification of these movies indicated that P-Rex1 drove a modest, but significant difference in migration speed, where mean velocity was increased from 0.354 µm min^−1^ to 0.415 µm min^−1^ (Figure 2B). Importantly, the expression of P-Rex1 resulted in a marked (1.7 fold) increase in forward migratory index (FMI) (Figure 2C), a measure of migration perpendicular to the wound edge, and a 1.5 fold increase in migratory persistence (Figure 2D). Conversely, expression of Tiam1, although able to drive ruffling and cell shape changes (Figure 1), did not alter migratory persistence or FMI of cell migrating into scratch-wounds (Figure 2). Moreover, the GEF-dead mutant of P-Rex1, P-Rex-GD, did not alter any of these quantitative indices of cell migration (Figure 2).

**Figure 3:**
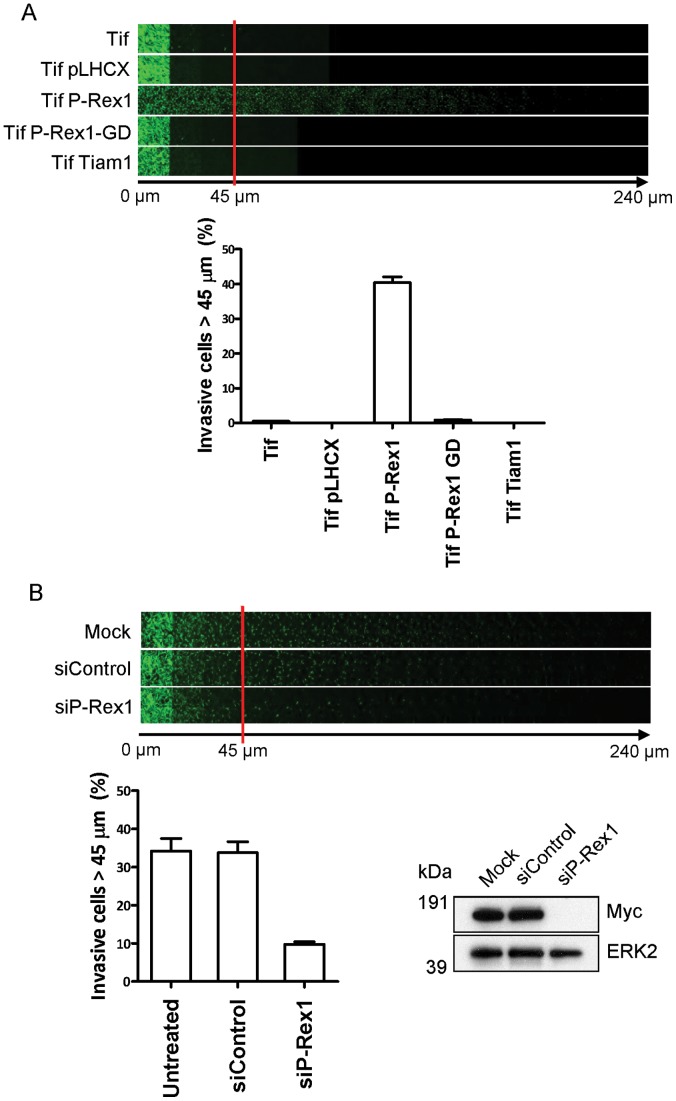
Expression of P-Rex1 promotes invasive behaviour of human fibroblasts. (A) Invasive migration of parental (Tif) and control vector (Tif pLHCX) orRacGEF expressing immortalised human fibroblasts into Matrigel plugs was determined by inverted invasion assay. Serial optical sections of Calcein-AM stained invasive cells were captured at 15 µm intervals by confocal microscopy and depicted as a montage of successive sections at increasing depth from left to right, as indicated. Invasive migration was quantified by measurement of fluorescence intensity of cells penetrating the matrix to a depth of 45 µm and beyond, and expressed as a percentage of the total fluorescence intensity of all cells within the assay. (B) Invasive migration of P-Rex1 expressing immortalised human fibroblasts transfected with non-targeting control or P-Rex1-specific siRNA oligonucleotides, with quantification as above (±SEM, ***P<0.0001 Unpaired t-test, 3 independent experiments).

P-Rex1 expression has been associated with tumour cell invasion and progression to metastasis in a number of cancer models *in vivo*. We have, therefore, investigated whether P-Rex1 overexpression was able to alter invasive-type migration of fibroblasts into plugs of Matrigel. Strikingly, while parental fibroblasts or those expressing a control vector did not invade towards a chemoattractant gradient of serum, more than 40% of P-Rex1-expressing cells migrated a distance of more than 45 µm into Matrigel, whereas P-Rex1-GD was ineffective in this regard (Figure 3A, B). Moreover, transient knockdown of P-Rex1 with siRNA oligonucleotides reduced invasion into Matrigel by at least 4-fold (Figure 3C, D). Moreover, P-Rex1-driven invasiveness was dependent upon GEF activity, as P-Rex1-GD expressing cells were unable to invade into Matrigel under identical conditions (Figures 2 and 3). Importantly, the related RacGEF Tiam1 was unable drive invasion of fibroblasts into Matrigel (Figure 3A). These data indicate that while P-Rex1 and Tiam1 are capable of increasing cellular ruffling and altering cell shape, only P-Rex1 is linked to the cell’s migratory machinery in a way that drives cell migration in both 2D and 3D contexts.

### P-Rex1 Driven Morphological Changes and Cellular Migration Requires a Factor that is Present in Serum

**Figure 4:**
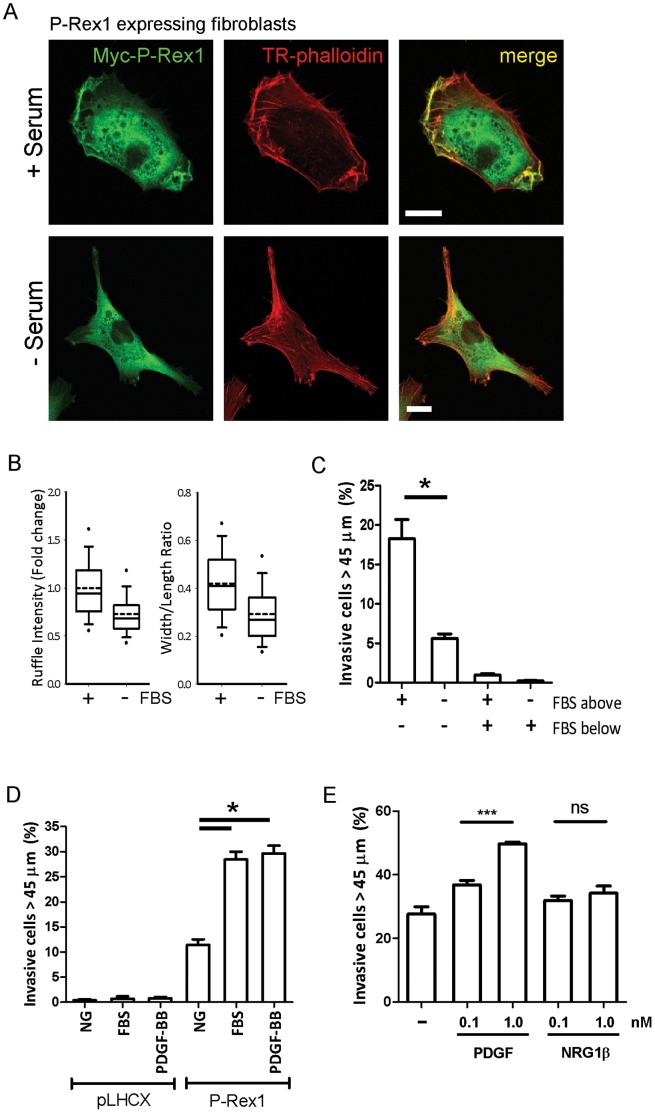
P-Rex1 requires a factor present in serum in order to drive alterations in cell shape and invasion; PDGF can substitute for serum to drive invasion of P-Rex1 expressing cells. (A) P-Rex1 expressing fibroblasts cultured under normal growth conditions or serum-starved for 12 hours were labelled as for Figure 1A above. (B) High throughput imaging was used to quantify the ruffle intensity and mean cellular width/length ratio of P-Rex1 expressing fibroblasts in the presence and absence of serum. (C) The requirement for a serum gradient in the invasive migration of P-Rex1 expressing fibroblasts was determined using an inverted invasion assay as for Figure 3A. (D) The invasive migration of vector control (pLHCX) or P-Rex1 expressing fibroblasts into Matrigel in the absence or presence of a serum (0–10%) or PDGF-BB (0–50 ng ml^−1^) gradient was assayed and quantified as for Figure 3B (±SEM, ***P<0.0005, Mann-Whitney rank sum test, 3 independent experiments). (E) The comparative invasive migration of P-Rex1 expressing fibroblasts into Matrigel in the absence or presence of gradients of PDGF-BB or NRG1 was assayed and quantified as in Figure 3B (±SEM, ***P<0.0005 Mann-Whitney rank sum test, 3 independent experiments).

In serum-containing culture conditions, overexpression of P-Rex1 drives changes in fibroblast morphology and migration. However, following serum-starvation, P-Rex1 expressing cells revert to a state reminiscent of control fibroblasts (Figure 4A). Indeed, high throughput imaging indicated that serum starvation of P-Rex1 expressing cells reduced the mean ruffle intensity to that of vector control cells (Figure 4B), and the mean cellular width/length ratio was reduced by 1.4 fold, again to a value similar to vector control (Figure 4B). Consistent with this, the invasive potential of P-Rex1 expressing fibroblasts was dependent upon the presence of a chemoattractant serum gradient (Figure 4C). Indeed, upon the imposition of a 10% gradient of serum across a plug of growth factor-depleted Matrigel, approximately 18% of P-Rex1 expressing fibroblasts penetrate more than 45 µm into the matrix. Conversely, if the serum concentration was the same both above and below the Matrigel plug, or if the gradient was reversed then P-Rex1 expressing cells were unable to invade into the Matrigel (Figure 4C).

We therefore sought to identify the serum factor responsible for supporting P-Rex1-dependent invasiveness. Many serum components that have previously been associated with RhoGTPase-driven cell migration (such as epidermal growth factor (EGF) and lysophosphatidic acid (LPA)) did not support the invasiveness of P-Rex1 expressing fibroblasts when applied as a gradient across a plug of Matrigel (not shown). However, a gradient of PDGF-BB (0–50 ng/ml) was equally capable to drive the invasion of P-Rex1 expressing fibroblasts into Matrigel as was a 0–10% gradient of serum (Figure 4D). Importantly, a PDGF gradient had little effect on control fibroblasts, suggesting that P-Rex1 and PDGF signalling collaborate to drive invasive migration. As recent reports have detailed an association between Neuregulin/ErbB signalling and P-Rex1 activation [12], [13], we sought to compare the ability of Heregulin/NRG1 and PDGF-BB to support P-Rex1-driven fibroblast invasiveness. In these experiments, 0–0.1 nM or 0–1 nM gradients of PDGF-BB drove 1.4 and 2.4-fold increases respectively in invasive migration. By contrast, the same concentrations of Heregulin/NRG1 did not drive P-Rex1-dependent invasion. (Figure 4E).

### A Physical Association between P-Rex1 and PDGFRβ

**Figure 5:**
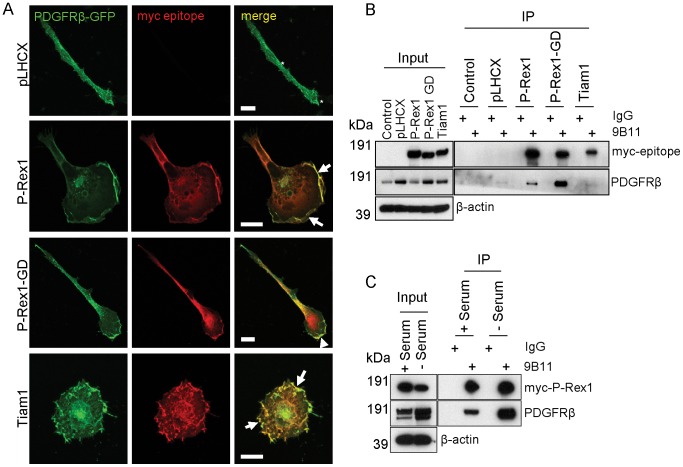
Association of P-Rex1 with PDGFRβ from immortalised human fibroblasts. (A) Control vector (pLHCX) or myc-RacGEF expressing immortalised human fibroblasts were transfected with GFP-tagged PDGFRβ and plated onto glass cover slips. Cells were fixed and the myc-tagged RacGEFs were stained by immunofluorescent labelling with an antibody recognising the myc-epitope followed by AlexaFluor-555 conjugated secondary antibodies. (B, C) Myc-tagged RacGEFs were stably expressed in immortalised human fibroblasts. The cells were then grown in serum-containing medium (B) or serum-starved for 18 hr (right hand lanes in (C)) prior to lysis in a buffer containing 0.15% Tween-20. Myc-tagged RacGEFs were immunoprecipitated from lysates using an antibody recognising the myc-epitope, and immunoisolated material was probed for the presence of PDGFRβ by immunoblotting.

Given the requirement for PDGF in P-Rex1 mediated invasion, we sought to confirm through a combination of confocal imaging and immunoprecipitation whether P-Rex1 and receptors for PDGF were physically connected. Fibroblasts expressing either a vector control or myc-tagged RacGEFs (myc-P-Rex1 or myc-Tiam1) were transiently transfected with a GFP-tagged fusion protein of the PDGF receptor β polypeptide (PDGFRβ) [17], and visualised by confocal microscopy. PDGFRβ-GFP colocalised closely with each of P-Rex1, P-Rex1 GD and Tiam1 in ruffling structures which are characteristic of active GEF expression (Figure 5A, arrows). It is intriguing that PDGFRβ and P-Rex1 GD also appear to co-localise, although limited regions of membrane ruffling which are located at the poles of the spindle-shaped P-Rex1 GD expressing cells (Figure 5A arrowheads).

To determine whether P-Rex1 and PDGFRs were associated physically with one another, we immunoprecipitated myc-tagged P-Rex1 and Tiam1 from fibroblasts and probed for the presence of the PDGFRβ. This revealed that PDGFRβ associated with P-Rex1, but not with Tiam1 (Figure 5B). Moreover, PDGFRβ was strongly associated with P-Rex1 GD indicating that P-Rex’s GEF activity was not required for this interaction (Figure 5B). Furthermore, P-Rex1 and PDGFRβ coimmunoprecipitated equally well in serum-starved or serum-replete cells indicating that activation of the receptor was not necessary for the complex to form (Figure 5C). Although we are able consistently to coimmunoprecipitate PDGFRβ with P-Rex1, we have found that a similar physical association between PDGFRα and P-Rex1 cannot be reproducibly demonstrated in these fibroblasts.

### P-Rex1 Mediated Invasion Requires PDGFRβ

**Figure 6:**
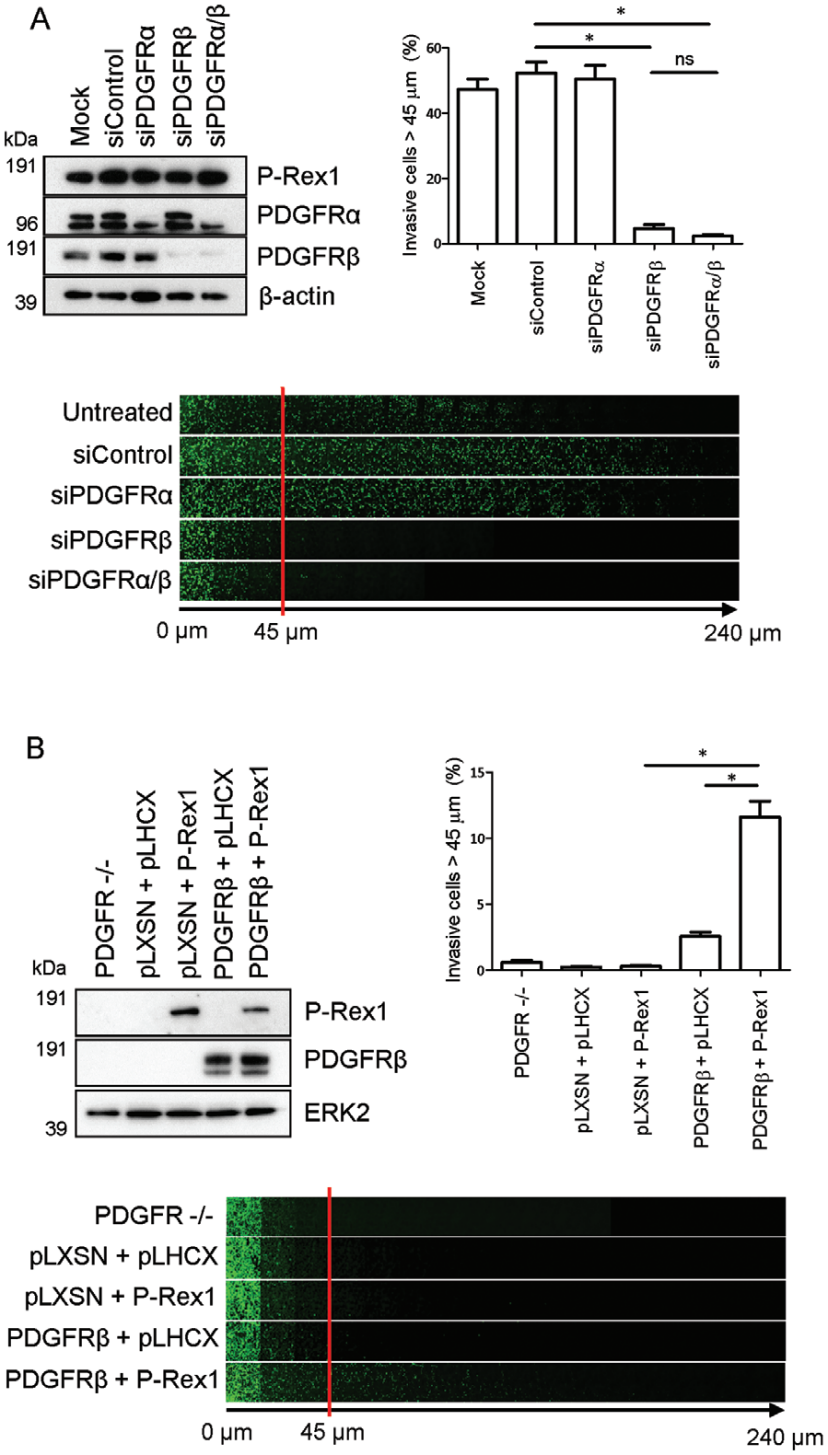
PDGFRβ is required for invasive migration of P-Rex1 expressing human fibroblasts. (A) Invasion of P-Rex1 expressing human fibroblasts transfected with non-targeting siRNAs (siControl) or those targetting PDGFRα or PDGFRβ,either alone or in combination, was assayed and quantified as for Figure 3. (B) Embryonic fibroblasts from PDGFRα/β −/− mice were stably transfected with P-Rex1 or PDGFRβ, either alone or in combination. Invasion was determined as for Figure 3. (±SEM, *P<0.05, **P<0.005 Mann-Whitney rank sum test, 3 independent experiments).

As PDGF-BB has high affinity for and can induce both homo- and heterodimerisation of the PDGF receptor polypeptides α and β [18], [19], we sought to examine the contribution of PDGFRα and PDGFRβ to P-Rex1 dependent migratory phenotypes in this cell type. Western blotting indicated that we were able to selectively suppress expression of PDGFRα and PDGFRβ either alone or in combination using siRNA (Figure 6A). siRNA of PDGFRβ strongly suppressed invasive migration of P-Rex1 expressing fibroblasts into Matrigel toward a gradient of serum, whereas knockdown of PDGFRα was ineffective in this regard (Figure 6A).

These findings were consolidated by rescue experiments with PDGFR null mouse embryonic fibroblasts, a cell culture model in which the genes for both PDGFRα and PDGFRβ have been disrupted [20], and which do not express endogenous P-Rex1 (Figure 6B). Initially, human PDGFRβ or a control vector were stably expressed in PDGFR −/− MEFs, which following several rounds of selection, were subjected to a further round of retroviral infection with human P-Rex1 or secondary vector control constructs. This approach yielded four cell lines stably expressing PDGFRβ or P-Rex1 either individually or in combination (Figure 6B), and allowed for the determination of the individual contribution of P-Rex1 and PDGFRβ to invasive migration in this model system. PDGFR −/− fibroblasts were poorly invasive and expression of P-Rex1 did not alter this. Moreover, expression of PDGFRβ in mouse fibroblasts that do not express P-Rex1 only drove invasion to a very limited extent. However, the combined expression of P-Rex1 with PDGFRβ dramatically increased the ability of mouse fibroblasts to invade into Matrigel (Figure 6B). Taken together, these data demonstrate a close spatial, physical and functional relationship between P-Rex1 and PDGFRβ in the migration of fibroblasts through 3D microenvironments.

**Figure 7:**
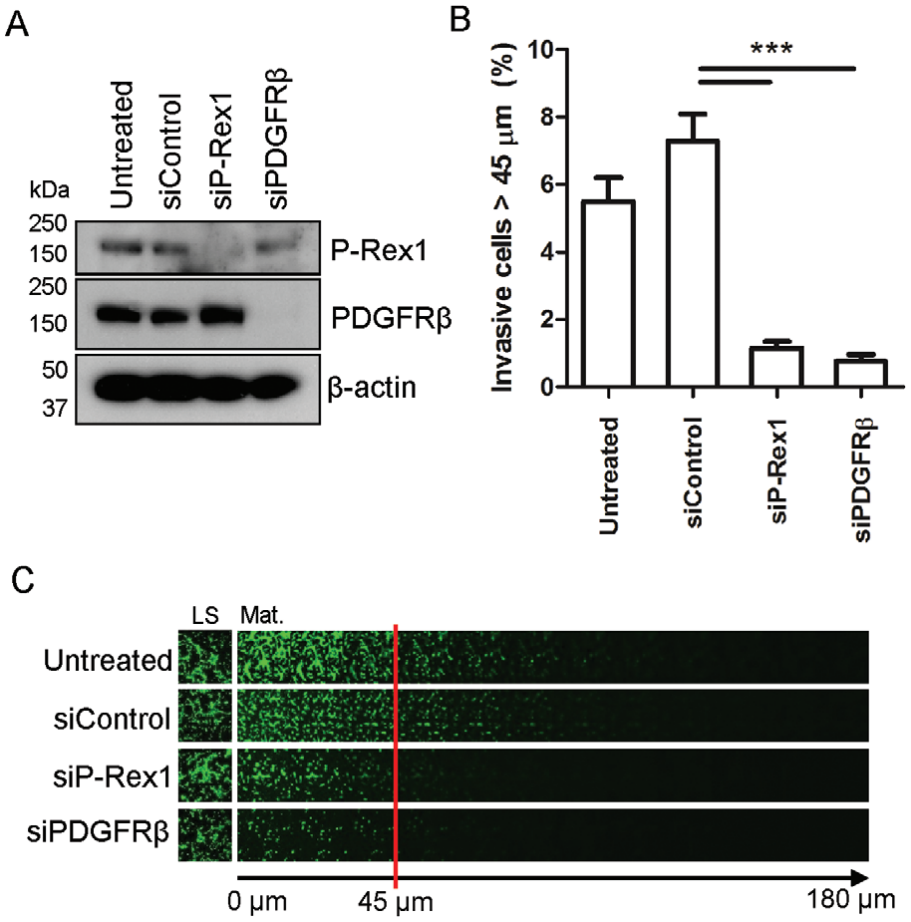
Expression of P-Rex1 and PDGFRβ are required for invasive migration of WM852 melanoma cells. (A) Immunoblot showing depletion of P-Rex1 or PDGFRβ following transfection of cells with non-targeting control or siRNA oligonucleotides directed against P-Rex1 or PDGFRβ. (B,C) Invasive migration of WM852 cells into Matrigel following transfection with siRNA oligonucleotides targeting P-Rex1 or PDGFβ was determined as for Fig. 3A. In (C), panels have been appended to demonstrate the adherence of the WM852 cells to the lower surface (LS) of the transwell.(±SEM, ***P<0.0005 Mann-Whitney rank sum test, 3 independent experiments).

Having described a functional and physical association between P-Rex1 and PDGFRβ in human fibroblasts, we next sought to assess the relationship between these proteins in a cell type that expresses them endogenously. As we have previously reported that P-Rex1 plays a key role in melanoma progression [21], we screened a number of melanoma cell lines for the combined expression of P-Rex1 and PDGFRβ. In this way we identified the WM852 melanoma line as one which expresses detectable levels of P-Rex1 and PDGFRβ and which is amenable to gene silencing with specific siRNA oligonucleotides (Figure 7A). siRNA of either P-Rex1 or PDGFRβ dramatically reduced the invasive migration of WM852cells into growth factor-reduced Matrigel plugs, when a gradient of 10% FBS supplemented with 5 ngml^−1^ PDGF-BB was used as a chemoattractant (Figure 7B, C).

### Inhibition of PDGFR or its Downstream Signalling through PI3K Perturbs P-Rex1 Mediated Migratory Phenotypes

Although we have found that disruption of PDGFRβ expression by siRNA silencing or genetic deletion opposes P-Rex1 mediated cellular invasion, it was clear that neither the presence of serum nor activation of the receptor was required for coimmunoprecipitation of P-Rex1 and PDGFRβ (see Figure 5C). We, therefore, addressed whether pharmacological perturbation of PDGFR activation would have similar effects upon P-Rex1 mediated migration and invasion as does siRNA or genetic depletion of the receptor.

**Figure 8:**
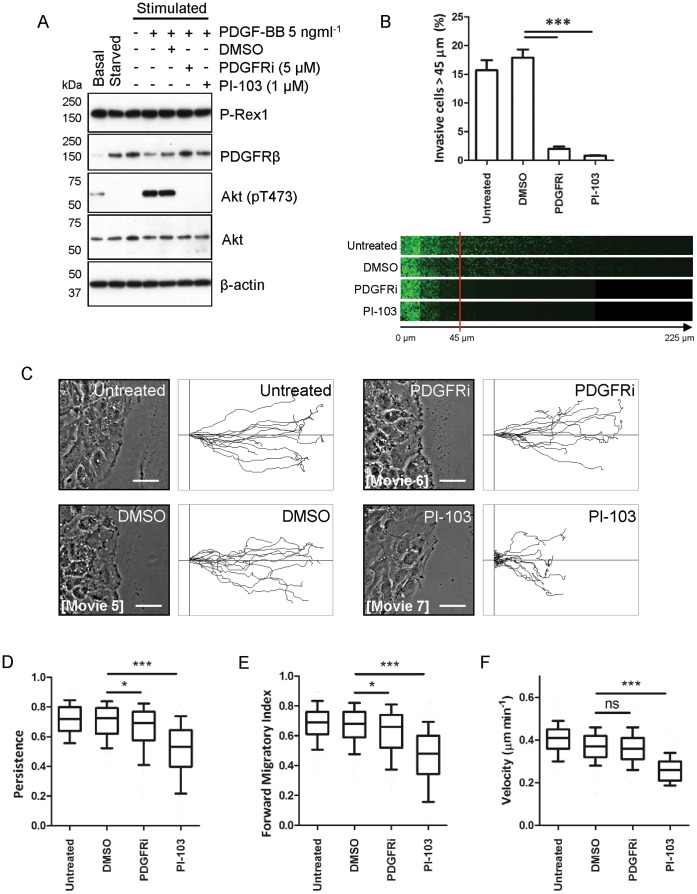
Chemical inhibition of PDGFR signalling alters the migratory behaviour of P-Rex1 expressing cells in both 2D and 3D environments. (A) Following a period of serum starvation, P-Rex1 expressing fibroblasts were stimulated with PDGF-BB, in the presence and absence of DMSO control or chemical inhibitors as indicated. Cells were the lysed and the levels of P-Rex1 and PDGFRβ were determined by Western blotting. The activity of the PI3K/Akt axis was determined by Western blotting for Akt which was phosphorylated at Thr^473^. (B) Quantification of the invasive migration of P-Rex1 expressing fibroblasts into Matrigel in the presence and absence of DMSO, PDGFRi (5 µM) or PI-103 (1 µM) as indicated (±SEM, ***P<0.0005 Mann-Whitney rank sum test, 3 independent experiments). (C) P-Rex1 expressing fibroblasts emerging from the migratory front of scratch-wound assays in the presence and absence of DMSO PDGFRi (5 µM) or PI-103 (1 µM) were analysed using time-lapse microscopy, with images captured every 10 minutes over a period of 18 hours. Individual cells were manually tracked and the migratory path of representative cells depicted as track plots. The phase contrast images correspond to the initial frame from the supplementary movies S5–S7 as indicated. (D-F) Influence of chemical inhibition on migratory behaviour was quantified as in Figure 2 by determination of mean cellular velocity (D), forward migratory index (E) and migratory persistence (F). (Bars 50 µm, *P<0.05, ***P<0.0005, Mann-Whitney rank sum test).

We found that treatment of P-Rex1-expressing fibroblasts with inhibitors of either PDGFR (PDGFRi; 5 µM) or PI3K (PI-103, 1 µM) was sufficient to oppose signalling downstream of PDGFR, as assessed by western blotting for phosphorylation of Akt at Thr^473^; a well-established read-out of activity of the PI3K signalling axis (Figure 8A). Having established the ability of these inhibitors to oppose PDGFR signalling, we determined their impact on the migratory behaviour of P-Rex1-expressing fibroblasts in scratch-wound assays. Pharmacological inhibition of PDGFRβ had no significant effect on migratory velocity, but PDGFRi had a small and statistically significant effect on the forward migratory index and migratory persistence (Figure 8C–D, supplementary movies S5 & S6). More strikingly, inhibition of the PI3K pathway with PI-103 significantly decreased the migratory velocity, forward migratory index and persistence of P-Rex1 expressing fibroblasts moving across plastic surfaces (Figure 8C–D, supplementary movies S5 & S7).

Subsequently, we examined the effect of inhibition of PDGFRβ or PI3K on invasion of P-Rex1-expressing fibroblasts through Matrigel. Inhibition of either the PDGFR family or PI3K strongly inhibited the ability of P-Rex1-expressing fibroblasts to migrate into Matrigel toward a gradient of FBS (0–10%). (Figure 8B). Taken together, these data indicate that the ability of P-Rex1 to drive both invasion into Matrigel and to support persistent on plastic surfaces requires the function of signalling pathways downstream of PDGFR.

## Discussion

P-Rex1 is an exchange factor for the RhoGTPase molecule Rac1, which has been implicated in progression to metastasis in a number of cancer models [11]–[14]. Through ectopic expression of P-Rex1 in cell culture models, we have identified a close physical and functional relationship between P-Rex1 and the receptor tyrosine kinase PDGFRβ during the acquisition of invasive cellular migration *in vitro*.

P-Rex1 is a well-established upstream regulator of RhoGTPase signalling [6], [9], [22]. In our hands, as in prior reports, ectopic overexpression of the RacGEF P-Rex1 or the related molecule, Tiam1 results in morphological changes in immortalised human fibroblasts [6], [23], [24]. These phenotypic changes are characteristic of increased Rac1 activity [16], and suggest that expression of P-Rex1 or Tiam1 influences the balance of RhoGTPase signalling. Importantly, these alterations to cell shape could not be driven by expression of a catalytically inactive mutant P-Rex1 molecule (E56A, N238A), suggesting dependence upon inherent GEF activity. The morphological changes reported in this study are truly robust, as more than 7,500 cells per condition were assayed, utilising a high throughput imaging system and two independent morphological criteria. Thus, we can say objectively and with confidence that active P-Rex1 and Tiam1 drive morphological adaptation in human fibroblasts. Importantly, even when subjected to such detailed quantitative analysis, it is clear that both P-Rex1 and Tiam1 induce alterations to cell shape and ruffling that are indistinguishable from one an another, and entirely consistent with the ability of these two molecules to activate Rac.

When one analyses cell migration, the phenotypes induces by P-Rex1 and Tiam1 are quite distinct. In scratch-wound assays, P-Rex1 drove a significant, GEF-dependent increase in mean velocity, persistence and forward migratory index of cells at the migratory front of the wound [25]. However, no change in the migratory behaviour of Tiam1 expressing cells was observed, despite clear alterations in the morphology of sparsely cultured non-migrating cells. This implies that a generalised increase in RacGEF activity is not sufficient in itself to influence migratory behaviour and that P-Rex1 has a particular role in this regard. Consistent with previously described Tiam1 driven formation of Rac1 dependent cell-cell contacts [26], [27], we observe loss of refractile morphology of Tiam1 expressing fibroblasts under brightfield microscopy at high cell densities which hints at altered cell to cell adhesion (Figure 2A). Taken together, these studies indicate that different functions can be ascribed to the two Rac GEFs of the present study - with Tiam1 primarily influencing cell:cell adhesion, whilst P-Rex1 is a powerful promoter of cellular migration.

The earliest characterisation of P-Rex1 described a synergistic regulation through association with the phospholipid PI(3,4,5)P_3_ and βγ subunits of the heterotrimeric G-proteins [6], [9], indicating that it is potentially capable of responding to signalling inputs generated downstream of both G-protein coupled receptors and RTKs. Moreover, these studies indicate that P-Rex1 may be recruited to the plasma membrane by association with the PI(3,4,5)P_3_ produced by growth factor receptor signalling. This said, our observation of a physical association between P-Rex1 and an RTK (PDGFRβ) occurs in serum-starved cells indicates that regulated production of PI(3,4,5)P_3_ is not a prerequisite for P-Rex1 recruitment cellular membrane compartments. Additionally, P-Rex1-GD co-immunoprecipitates efficiently with PDGFRβ indicating that Rac activation and the cytoskeletal changes occurring downstream of this are not required for recruitment of P-Rex1 to PDGFRβ.

In addition to the physical association between P-Rex1 and PDGFRβ, there is also a close functional relationship that is not shared by Tiam1. Here we identify PDGF to be a serum component that is both necessary and sufficient for P-Rex1 driven migration and chemotaxis in a 3D microenvironment. Furthermore, we have used siRNA to show that this is mediated primarily via PDGFRβ and does not require PDGFRα. Furthermore, in fibroblasts from mice in which the genes for PDGFRα and β have been disrupted, expression of PDGFRβ is sufficient to restore P-Rex1-driven migration towards a serum gradient. Taken together, these results indicate that PDGFRβ, and not PDGFRα is a major contributor to P-Rex1 “activation” in regard of invasive cell migration in the context of our culture model. Subsequent chemical inhibition of the PDGFR family in P-Rex1 expressing fibroblasts also demonstrates that an active signalling axis exists involving PDGFR and P-Rex1, whereby specific loss of PDGFR activity while maintaining protein expression, negatively affects P-Rex1-dependent migratory behaviour both on a 2D substrate and in 3D. Furthermore, similar observations made following inhibition of PI3K serve to highlight the importance of phosphatidyl inositide signalling in regulation of P-Rex1 activity.

There is a strong correlation between P-Rex1 expression and progression to metastasis in a number of cancer models[11]–[14]. The observation therefore, that expression of this single RacGEF was sufficient to drive a dramatic increase in invasive migration in 3D matrices *in vitro* was particularly striking. Furthermore, evidence exists to suggest that a functional relationship between P-Rex1 and PDGFRβ as described here may be a contributing factor to cancer progression *in vivo*. The first suggestion of an association arose in a genetic screen to identify key factors in glioma progression *in vivo*, which highlighted PREX1 as a gene which cooperates with PDGF signalling to promote metastasis [28], [29]. Moreover, in light of our recent description of P-Rex1 as a key driver of melanoma progression *in vivo* [14], it is noteworthy that a common mechanism of acquired resistance to novel therapeutic kinase inhibitors in BRAF^V600E^ driven melanoma is upregulation of PDGFRβ [30], [31]. Indeed, we present data which demonstrates that *in vitro* invasive migration of a human melanoma derived cell line, WM852, is dependent upon expression of both P-Rex1 and PDGFRβ.

In addition to their contribution to malignant growth in certain cancer cell types, the PDGF receptors and autocrine PDGF signalling are critical drivers of recruitment and growth of non-transformed cells, such as pericytes, vascular smooth muscle cells and stromal fibroblasts to the tumour site. These can in turn contribute both to tumour growth and vascularity. It is therefore important to note that through the *in vitro* experimentation detailed here, we examine tumour cell autonomous effects of PDGFR stimulation, in particular in the context of P-Rex1 mediated cellular migration, and do not consider the role of the PDGF signalling axis on survival or growth of stromal cells in an extant tumour *in vivo*. By way of example, while silencing of PDGFRβ negatively affects *in vitro* migratory behaviour in individual cell lines, a number of publications have indicated that mono- or combination therapies incorporating inhibition of PDGFRs *in vivo* can result in a more aggressive invasive or metastatic disease [32]–[35]. This appears at least in part due to a stromal response, where negative effects upon both pericyte growth and migration, can lead to poor pericyte coverage on nascent blood vessels, resulting in a “leaky” tumour vasculature, more susceptible to intravasation [33], [35], [36] Moreover, such therapies may have a similar systemic effect, resulting in a more conducive vascular environment for extravasation and subsequent metastasis at a distant site [32].

It seems that the relationship between P-Rex1 and PDGFRβ may have direct relevance to tumour progression, and further studies to reveal the details of the complex formed between these two important signalling proteins and how they collaborate to direct cell migration will be necessary to determine whether components of this pathway can be targetted to oppose cancer progression and metastasis.

## Materials and Methods

### Cell Culture

hTERT immortalised human foetal fibroblasts (Tif cells) [37] and PDGFR nullizygous mouse embryonic fibroblasts (“F” cells) [20] and their derivatives were maintained in DMEM (Invitrogen Life Technologies Ltd.) supplemented with 10% foetal calf serum (Biowest S.A.S.) and 200 µM L-Glutamine. “F” cells were the kind gift of Prof. Andrius Kazlauskas of the Schepens Eye Institute, Boston. WM852 melanoma cells were maintained as previously reported [38], and were the kind gift of Professor Lional LaRue of the Institut Curie, Orsay, Paris.

### Stable Overexpression of P-Rex1/Tiam1 Constructs

pcDNA3.1-myc-Tiam1 was the kind gift of Dr. Angeliki Maliri (Paterson Institute for Cancer Research, Manchester, UK). pLNCX2-PDGFRα and pLXSN-PDGFRβ were the kind gift of Professor Andrius Kazlauskas (Schepens Eye Research Institute, Boston, USA). Stable cell lines expressing exogenous PDGFRβ, myc-epitope tagged human P-Rex1 or Tiam1 constructs were generated by retroviral infection using the modified Retro-X retroviral expression system (Clontech Ltd.). High-titre, replication-incompetent retroviral particles encoding the mRNA of interest were produced in either Phoenix Ampho or Eco packaging lines (Orbigen) as appropriate, with transfer the coding region of interest, along with a selectable marker achieved by subsequent infection of target lines. Pooled cell lines stably expressing the construct of interest were isolated by selection with the appropriate antibiotic over multiple passages. Faithful expression of the protein of interest was determined by immunodetection with both epitope tag specific and protein specific primary antibodies.

### Antibodies, Drugs and Recombinant Proteins

Rabbit polyclonal antibody raised against human P-Rex1 (HPA001927) and mouse monoclonal antibodies specific to β-actin (clone AC-15, A1978) and β-tubulin (clone TUB2.1, T4026) were obtained from Sigma-Aldrich Ltd. Mouse monoclonal antibodies specific for the Myc-epitope tag (clone 9B11, 2276S) and PDGFRβ (clone 2B3, 3175S) were obtained from Cell Signalling Technology/New England Biolabs UK. PDGFR inhibitor III (521232) was obtained from Merck and PI-103 (2930) was from Tocris Bioscience. Recombinant human EGF (AF-100-15) was obtained from Peprotech EC Ltd, recombinant human PDGF-BB (P3201), and NRG1 (SRP3055) were obtained from Sigma-Aldrich Ltd.

### siRNA Treatments

Transient knockdown of target proteins was achieved through consecutive rounds of liposome-mediated transfection with the appropriate siRNA oligonucleotides, 48 hours apart. Liposomal transfection reagent (301702, HiPerFect, Qiagen Ltd.), non-targetting control oligonucleotides (1027281, AllStars Negative Control, sequence not provided), P-Rex1 specific (SI00692391, Hs_PREX1_1_HP, CCACATGATGATGAACAAGAA) and Rac1 specific (SI02655051, Hs_RAC1_6_HP, sequence not provided) oligonucleotides were obtained from Qiagen Ltd. Oligonucleotides specific for PDGFRα (D-003162-09, GGCCUUACUUUAUUGGAUU), PDGFRβ (D-003163-06, GGAAUGAGGUGGUCAACUU) and an appropriate non-targetting control (D-001810-10) were obtained from Dharmacon Ltd. Faithful silencing of the appropriate protein of interest was determined by immunoblotting. Briefly, cells were lysed on ice in 50 mM Tris-HCl pH 6.8, 10% (v/v) glycerol and 1% (w/v) SDS, supplemented with protease inhibitor (#78410, ThermoScientific) and phosphatase inhibitor (P2850, Sigma-Aldrich Ltd.) cocktails. Cell lysates were passed through a 26-gauge needle three times and cell debris removed by centrifugation at 10,000×g and 4°C for 10 minutes. These lysates were then denatured by boiling for 10 minutes in LDS buffer (Invitrogen Life Technologies Ltd.) under reducing conditions. Proteins of interest were then resolved by SDS-PAGE and detected with specific antibodies.

### Scratch-Wound Assay and Timelapse Microscopy

Confluent monolayers of fibroblasts were seeded onto 6-well tissue culture dishes and linear scratches made with a sterile 200 µl pipette tip. These cells were incubated under normal tissue culture conditions until cells at the wound edge began to migrate (∼ 4 hrs), and timelapse images were captured every 10 minutes for 18 hours. Imaging was performed with the X10 objective (Axiovert S100, Carl Zeiss MicroImaging, Inc) in a controlled atmosphere of 5% CO_2_ at 37°C. Cell tracking was performed with the *Manual Tracking* plugin for the ImageJ software platform and mean velocity, persistence and forward migratory index were calculated with the associated *Chemotaxis and Migration Tool* plugin (http://rsb.info.nih.gov/ij/).

### Inverted Invasion Assay

Inverted invasion assays were performed as previously described [39]. In brief, Matrigel protein matrix (BD bioscience) was allowed to polymerise in Transwell permeable inserts (Corning Ltd.) over 30 minutes at 37°C. The inserts were inverted and cells seeded directly onto the filter surface. After 3 hours at 37°C, the inserts were placed in serum-free tissue culture medium in 24-well plates, and media containing the appropriate chemoattractant placed above the Matrigel matrix. In the case of siRNA mediated transient knockdown experiments, invasion assays were prepared 24 hours after the second round of lipofection. In the case of inhibitor studies, drug or DMSO was included at the appropriate concentration in both chemoattractant and serum-free media, and as a component of the Matrigel matrix. Furthermore, in these studies inserts were transferred to fresh serum-free tissue culture medium containing the appropriate drug or control at 24 and 48 hours. In all assays, at 72 hours post-seeding, invasive cells which had entered the Matrigel were stained with the fluorescent live-cell dye Calcein-AM, visualised through confocal microscopy of optical sections obtained in the z-plane at 15 µm intervals, and quantified with the ImageJ *Area Calculator* plugin.

### Immunoprecipitations

Human fibroblasts expressing constructs of interest were lysed in IP-lysis buffer (200 mM NaCl, 75 mM Tris-HCl pH 7, 7.5 mM EDTA, 7.5 mM EGTA, 0.15% (v/v) Tween-20, supplemented with protease and phosphatase inhibitor cocktails. Cell lysates were cleared as described above. Dynal magnetic beads conjugated to sheep anti-mouse IgG (Invitrogen Life Technologies Ltd.), pre-blocked with a solution of 1% (w/v) BSA in PBS, were bound to anti-myc epitope or isotype matched control monoclonal (IgG2a, Sigma) antibodies. Antibody bound beads were subsequently incubated in 1 ml of a 0.25 mgml^−1^ lysate solution for 2 hours at 4°C with constant rotation. Unbound proteins were removed from the beads by extensive washing in lysis buffer, and specifically associated proteins were eluted through incubation in lysis buffer supplemented with 1% SDS for 10 minutes. Eluted proteins were resolved by SDS-PAGE and detected by immunoblotting as described above.

### Immunofluorescent Imaging of Fixed Specimens

Immunofluorescent staining of fixed cell specimens was performed as described previously [40]. Briefly, cells were seeded at the appropriate density onto coverglass, or in the case of high-throughput imaging, into individual wells of a glass-bottomed 24-well tissue culture plate (Iwaki Ltd.), 24 hours prior to fixation. Cells were washed in PBS, and fixed with a 30 minute incubation in a 3% (w/v) solution of paraformaldehyde in PBS, followed by permeabilisation with 0.25% (v/v) Triton-x-100 in PBS for 5 minutes, both at room temperature. Non-specific antibody binding was blocked with incubation in a 5% (w/v) BSA in PBS. Primary and secondary antibody incubations were carried out in a 0.1% (w/v) solution of BSA in PBS for 1 hour each at room temperature, separated by 3×10 minute washes in a solution of 0.05% (v/v) Tween-20 and 150 mM NaCl in PBS. The antibody and drug concentrations used were as follows –9B11 anti-myc epitope, 1/1000 (0.8 µgml^−1^), AF488 conjugated goat anti-mouse secondary, 1/200 (10 µgml^−1^), TRITC-conjugated phalloidin, 1/40 (5 Uml^−1^) and DAPI 1/500 (100 ngml^−1^). Stained coverslips were applied to slideglass with Hydromount mounting media (National Diagnostics Ltd.), supplemented with 2.5% DABCO (Sigma-Aldrich Ltd.) as an anti-oxidant/anti-fade agent. Cells mounted on slideglass were analysed with a Zeiss LSM710 upright laser-scanning confocal microscope (Carl Zeiss MicroImaging Ltd.). Specimens prepared for high throughput imaging were stored in PBS at 4°C until needed.

### High-Throughput Imaging

Imaging and analysis was performed with a high-throughput Operetta fluorescent microscopy platform and Harmony image analysis software (Perkin Elmer Ltd). Briefly, fibroblasts were cultured in a multiwell-dish format and processed for immunofluorescent staining as described above. Fluorescent cells were detected through fluorescence intensity in either the A488 (AF488-anti-myc) channel, or A546 (TRITC-phalloidin) channel, and individual width/length ratio determined. Dorsal membrane ruffles present in an outer ring region defined at 20% of the distance from the cell edge to its centre were detected using a texture analysis algorithm, whereby fluorescence intensity in ruffle structures was again detected in either the A488 (AF488-anti-myc) channel, or A546 (TRITC-phalloidin) channel and subsequently normalised to background fluorescence (i.e. fluorescence not ascribed to ruffles).

## Supporting Information

Movie S1 **Movement of 1 pLHCX vector control expressing fibroblasts into a scratch-wound.**
(MOV)

Movie S2 **Movement of pLHCX-myc-P-Rex1 expressing fibroblasts into a stratch-wound.**
(MOV)

Movie S3 **Movement of pLHCX-myc-P-Rex1 GD expressing fibroblasts into a scratch-wound.**
(MOV)

Movie S4 **Movement of pLHCX-myc-Tiam1 expressing fibroblasts into a scratch-wound.**
(MOV)

Movie S5 **Movement of DMSO-treated human fibroblasts into a scratch-wound.**
(MOV)

Movie S6 **Movement of PDGF receptor inhibitor (PDGFRi III, 5 µM)-treated human fibroblasts into a scratch-wound.**
(MOV)

Movie S7 **Movement of PI-103 (1 µM)-treated human fibroblasts into a scratch-wound.**
(MOV)
